# Integrating play-based pedagogy into a knowledge-based curriculum: supporting children’s understanding of anger in a Chinese kindergarten

**DOI:** 10.3389/fpsyg.2025.1673016

**Published:** 2025-10-15

**Authors:** Qiming Liu, Helen Demetriou

**Affiliations:** Faculty of Education, University of Cambridge, Cambridge, United Kingdom

**Keywords:** anger, early childhood education, emotions, play-based pedagogy, cultural-historical theory

## Abstract

**Introduction:**

We examined whether culturally adapted play can support anger understanding within knowledge‑based Chinese kindergarten curricula.

**Methods:**

One‑week plan with 40 children (5–6 years) and four teachers; mixed play types across five MoE domains. Outcomes: oral assessment by domain and teacher interviews; descriptive/thematic analyses.

**Results:**

Strengths in language, social, arts and health; between‑class variability. Teachers noted richer emotion vocabulary, listening and peer problem‑solving; some misconceptions persisted.

**Discussion:**

Teacher‑centred, culturally appropriate play can be embedded but needs teacher development; longer, controlled evaluations are recommended.

## Introduction

It has long been recognised that the early years plays a crucial role in an individual’s socio-emotional development (Gallagher et al., 2007; Rahmaniya et al., 2025). Studies have sought to explore empathy and emotions in diverse and Indigenous societies (Brebner et al., 2015; Humphries et al., 2018) due to the importance of all children acquiring empathy skills (Usta and Sahin, 2022; Wong and Power, 2024), improving levels of emotional intelligence (Rahmaniya et al., 2025), and supporting social interactions (Park et al., 2016). Furthermore, children who can regulate their emotions are likely to attain better academic outcomes than children who cannot (Bierman et al., 2008).

Studies from different contexts have explored the importance of developing children’s socio-emotional skills. These range from Hong Kong (Wong and Power, 2024), the United States (US) (Bierman et al., 2008; Humphries et al., 2018), South Korea (Park et al., 2016), Turkey (Usta and Sahin, 2022), and Australia (Brebner et al., 2015). In China, Meng et al. (2022) explored emotional regulation with collective play and cultural-historical theory in a state-run orphanage, finding that a culturally appropriate play plan in China could support a child’s regulation of fear. Given that play is an integral component of early childhood pedagogy, advocated strongly by Vygotsky (1987) and Piaget (1983) in the West, the merge of theories surrounding play, emotional development, and cultural-historical theory forms the basis of this present study.

In China, it is more common to focus on socially inappropriate behaviours and how these behaviours impact other people, rather than focus on children’s innate emotional states (Cheah and Rubin, 2004). Thus, when angry children’s behaviour (such as physical aggression) is immediately managed by the adult, but not further explored, the emotional state that led to the behaviour is not considered (Nelson et al., 2002; Wong and Power, 2024). Therefore, this article places this issue at the root of its aim: to consider how to create a culturally appropriate play-based pedagogy for use in China’s educational systems to develop children’s knowledge and understanding of anger. It presents a week-long play-based pedagogy plan which combines elements of knowledge, play, cultural-historical theory, and emotional development with the specific intention of ensuring that children are equipped with the skills to manage their emotions and feelings of anger.

### The importance of play

The broad nature of play demonstrates it is a useful pedagogy that provides a diverse array of learning experiences, child agency, and adult intervention. For example, play-based pedagogy features different play types, such as free play (child-led), guided play (child-led and adult scaffolded), and games (adult designed and scaffolded, featuring rules) (Zosh et al., 2017). While teachers’ decisions about children’s play experiences are impacted by cultural-historical, cognitive developmental, post-developmental and anthropological factors (Samuelsson and Carlsson, 2008; Blaise et al., 2014; McLean et al., 2022), the consensus among researchers clearly acknowledges the role of play-based pedagogy in the enhancement of children’s learning and developmental outcomes (Whitebread et al., 2017; Zosh et al., 2017; Lake et al., 2022). Therefore, in such a Confucian and adult-led learning context as China, adult designed and directed games have the potential to be used in the research setting, thereby reflecting a cultural-historical view of play-based pedagogy in this context.

When focussing on children’s developmental outcomes, play can help children to manage and express their emotions and cope with social situations in a safe, supportive environment (Vlaicu, 2014; Hart and Booth, 2017). Play allows children to experiment with different social roles, practise their empathy, and develop emotional regulation skills. Interactive peer play can facilitate children’s emotional experiences and support their ability to recognise other people’s desires and perspectives (Futterer et al., 2022). However, in China, children are discouraged from taking part in therapeutic play, therefore showing a barrier to accepting the role of play in supporting children’s emotional and mental health development (Cao, 2023), something which needs to be addressed when supporting anger management.

### Current practice in China

In 2001, China’s Ministry of Education (MoE) promoted a child-centred play-based pedagogy (MoE, 2001, 2012); however, effective practical implementation is limited (MoE, 2012; Yang, 2013). For example, teachers remain uncertain about what constitutes play-based pedagogy and how to implement it (Zhu and Zhang, 2008). This vagueness leads to inconsistent approaches which are influenced by Confucianism, meaning that many educators default to teacher-led, knowledge-oriented methods (Zhu and Zhang, 2008).

Zhu and Zhang’s (2008) research exemplifies the impact of “A hybrid of three cultures” (p. 175), consisting of Confucianism, Communist Culture and Western culture. Confucianism emphasises academic achievement, respect for authority, and the moral development of children through structured learning (Leung, 1998). As a result, play is viewed differently from Western countries, echoing Gosso’s (2009, p. 80) view that, “Each culture sees play in a distinct way.” In China, this manifests as an emphasis on academic pursuits (Shen, 2016; Cao, 2023). This is also true for parents, who tend to prioritise academic preparation for primary school and expect high levels of academic development (Peng, 2019), viewing play as secondary to knowledge acquisition (Xie and Li, 2018). Some parents even view play as a distraction from learning (Hu et al., 2016). Moreover, researchers have discovered that Chinese parents tend to prioritise ECE which entails teacher direction, carefully planned lessons with an academic focus, and highly structured instructional formats (Li and Rao, 2005; Tobin et al., 2009). Thus, cultural tension arises between the adoption of a Western-centric pedagogy in China, even though play has been accepted as a useful pedagogy for child development (Russ, 2003; Ginsburg et al., 2007).

Confucianism leads to the adoption of a curriculum that centres around building knowledge through previous knowledge (Yang, 2013). A Western constructivist would develop such knowledge through active learning and interactions with the environment and social context (Podolskiy, 2012), broadening the scope of learning from passive to active. Vygotsky’s (1987) socio-cultural theory suggests that children learn best in collaborative settings where they can engage with both peers and adults to develop social, cognitive, and emotional skills. This tension between structured, teacher-directed knowledge transfer and play-centred learning which encompasses a range of play types, from structured to unstructured, and teacher-led to free play (Zosh et al., 2017), is central to understanding how a knowledge-based curriculum can integrate play-based methods to support emotional development.

Communist ideology emphasises collective activities and group cohesion, focusing on structured, goal-oriented learning, memorisation, collective activities and discipline (Leung, 1998; Huang, 2006) to foster a sense of collective honour in students (Suo, 2022; Luo, 2023). When children meet these learning goals, play is often used as a teacher-directed tool for behaviour management (such as a reward) (Qiu, 2011). Moreover, even though game play is sometimes used in China, meaning that teachers plan specific activities with rules and structure, its effectiveness is impacted by teachers’ knowledge (Cai, 2021). This demonstrates the culturally specific context in which this article is positioned, calling for the development of a culturally specific play plan rather than simply using a play-based pedagogy plan developed in other contexts. As Tan and Chua (2015) state, a direct transfer of Western play-based pedagogy to China is unlikely to succeed, largely due to cultural differences.

One of the primary obstacles to implementing play-based pedagogy in China is the lack of teachers’ professional development and issues with effective policy implementation (Dong, 2013). Many teachers are more familiar with traditional, knowledge-based instruction which emphasises memorisation and teacher authority, again representing the influence of Confucianism (Leung, 1998). Even when professional development opportunities occur, such as through providing space for teacher collaboration, barriers to success emerge due to the strict hierarchical nature of the Chinese kindergarten administration system, which results in a lack of collaboration between teachers (Wang, 2006; Wang et al., 2023). Indeed, most teacher collaboration that takes place in China’s schools represents an institutional mandate as the result of a top-down management structure (Chen, 2006; Zhang and Pang, 2016). Hence, this article’s empirical study seeks to overcome this issue by providing an informal, reflective space in which teachers can collaborate in the planning process. Given that collaboration is recognised as an important aspect due to its links to motivation (Kolleck, 2019), perceptions of the school environment (Sawyer and Rimm-Kaufman, 2007), and ongoing professional improvement (Pugach and Johnson, 2002), this is an important aspect to include when working with teachers to create a novel play-based pedagogy framework.

Classroom management and assessment are other concerns. Large class sizes result in kindergartens opting for whole-group teaching rather than utilising play activities (Zhu, 2007; Wu et al., 2012). Furthermore, in terms of assessing play, this is usually an adult-developed approach which seeks to assess children’s school ‘readiness’, developmental milestones, or academic attainment (Meisels et al., 1993). In terms of play-based pedagogy, assessment can take the form of multiple methods depending on the aspect being assessed, including standardized tests to assess children’s social pretend play (Jaggy et al., 2020), using children’s mark-making created during play sessions to assess their literacy skills (Friedrich and Stagg, 2022), and direct observations of children’s play (Pellegrini, 2001). Indeed, observations are a common form of assessment in the early years and are sometimes complemented by checklists or observation instruments (Pellegrini, 2001); however, in the Chinese context, teachers do not know how to effectively use observation, and many kindergarten managers may not attach importance to observation (Shu, 2022). Based on this lack of observation, no clear guidelines are available to assess the outcomes of play-based learning; the MoE’s (2022) tools for kindergartens do not provide information about how to measure progress or attainment in social and emotional learning. In China, ECE remains heavily focused on academic outcomes, and there are no widely accepted methods for evaluating the socio-emotional benefits of play. The existing assessment frameworks, such as those in the “Ministry of Education Notice on the Issuance of Guidelines for Assessing the Quality of Kindergarten Care and Education” (MoE, 2022), focus on institutional factors like safety and teacher qualifications; they overlook how to measure children’s emotional and social development through play-based activities. This means that anger is not included in the framework. This is also represented in the ECE provincial government textbook “Shandong Province Kindergarten Curriculum Guidance” (Yu, 2013), where again, knowledge is prioritised over skills. Nonetheless, anger management must be viewed as a critical aspect of ECE, particularly in a collective society like China, where social cohesion and harmony are highly valued. Developing the ability to regulate emotions is essential for children’s long-term success, especially in terms of academic attainment (Garner, 2010; Djambazova-Popordanoska, 2016; Adynski et al., 2024), and play offers a unique opportunity to foster these skills (Lorimier et al., 1995; Hyvonen, 2011).

### The focus on anger

Young children’s emotional development and empathy acquisition are essential (Simon and Nader-Grosbois, 2023), and within the concept of empathy, anger plays a pivotal role. As Roberts and Strayer’s (1996) study found, emotional expressiveness and anger serve as key predictors of empathy. Empathy and anger impact children’s social behaviours, level of aggression, and relationships (Hoffman, 1977; Thompson, 1994; Roberts and Strayer, 1996). Hence, by specifically focusing on anger and children’s abilities to recognise and manage anger, we can support children’s wider empathy development, which in turn, can reduce instances of conflict (Coie and Dodge, 1998).

Forming part of human emotions, anger can act as a barrier to people’s empathetic responses, and children with high levels of empathy tend to be less angry (Eisenberg et al., 1992; Strayer and Roberts, 2004), have stronger academic, emotional, and social outcomes (Eisenberg and Fabes, 1998; Denham, 2006; Lemerise and Dodge, 2008). Liu et al. (2018, p. 2090) explain that “absolute levels of anger typically peak in early childhood and diminish as children become socialized and better able to regulate emotions.” For young children, anger is often the result of a blocked goal and serves as a medium for self-defence and the overcoming of such obstacles (Lemerise and Dodge, 2008). Developmentally, children’s levels of anger increase from the age of 1 year old as adults exert higher levels of control as they become toddlers (Roben et al., 2012). Due to their improved emotional regulation, it is expected that children’s levels of anger decrease in middle childhood (Denham et al., 1995).

Strayer and Roberts (2004) found that the relationship between anger and aggression is complex despite a clear, strong correlation between the two. They note that self-regulation and empathy can reduce instances of aggression, highlighting the importance of exploring anger and empathy in Early Childhood Education (ECE) (Strayer and Roberts, 2004). From a gender perspective, Chapman et al. (2006) note that school-aged girls have higher levels of empathy than boys, while Lawrence et al. (2015) similarly share that this is also true for girls’ abilities to read and understand non-verbal cues. Studies have also extended to empathetic behaviours towards animals and pets, whereby girls are significantly more empathic than boys (Daly and Morton, 2006). This continues into adulthood as females are, on average, more empathetic toward animals than their male counterparts (Pifer et al., 1994). Furthermore, the impact of boys having higher levels of anger than girls can manifest into hegemonic masculinity, whereby male bullying behaviours encompass anger and physical dominance (Rosen and Nofziger, 2019). Leppänen and Hietanen (2001) consider reasons for such differences in empathy, arguing that girls’ empathy may be higher than boys’ empathy due to social adjustment.

Wong and Power’s (2024) research which determined the impact of the ECE-Peace Ambassador project (a social emotional learning programme) on 4-5-year-old children’s emotional intelligence, prosocial and aggressive behaviour, and peer exclusion in the Chinese context drew on previous studies (such as Vitaro and Brendgen, 2012) to share that while aggression is a key concern among teachers (Li et al., 2016), such programmes can be beneficial in supporting children’s social emotional learning. Concerns surrounding child aggression are also present in Hong Kong and Mainland China, manifesting as bullying and self-reported problem behaviours among children (Fung and Tsang, 2006). In practice, this manifests as physical aggression or social threats, such as excluding another child from a group (Nelson et al., 2002; Wong and Power, 2024). In China, the authoritarian style of parenting, possibly due to the cultural influence of Confucianism (Xu et al., 2005), provides a context in which children experience a different style of parenting from their Western counterparts (Pearson and Rao, 2003). Given that most studies that have considered aggressive behaviour in children have been conducted in the West, Chan (2009) explored the link between authoritarian parenting and children’s aggression, finding that authoritarian parenting leads to children acquiring negative coping strategies and aggression. Although this article does not consider parenting styles in depth, the impact of culture here brings to light the impact of cultural-historical theory and the importance of recognising children’s experiences at home when seeking to support children with their understanding of anger in the classroom.

When creating a plan for children that supports their recognition and management of anger, this article recognises recent findings from Farley’s (2024) exploration of young people’s experiences and definitions of anger and aggression. In the study, 27 young people with an average age of 14.1 years shared their view that emotional expression is “a learned process… in which progress or change can be created” (Farley, 2024, p. 147). Hence, if anger can be learned, we can teach children from a young age how to manage and respond to these feelings.

### The present study

While aggression and self-regulation have been studied by Chan (2009), alongside Meng et al.’s (2022) exploration of play, cultural-historical theory and fear in a Chinese orphanage, research has not specifically considered the development of a culturally appropriate play plan for anger recognition and management in kindergartens. This present study therefore aims to fill this gap.

The data collection in this article took place in China after the COVID-19 pandemic, meaning that many of the children who took part in the study were in lockdown during their very early years. Furthermore, the one-child policy was in place until 2015, the two-child policy in 2016 and the three-child policy in 2021, meaning that these same children were also limited in terms of sibling interaction (Attané, 2022), impacting their emotional, language and social development (Rodriguez-Monge et al., 2023). Problematically, children’s levels of anger have emerged as a result of the pandemic (Tiwari et al., 2021).

This study adopts a theoretical framework that is based largely on cultural-historical theory (Vygotsky et al., 1997; Podolskiy, 2012). Vygotsky et al. (1997) state that cultural-historical theory is crucial in the formation of cultural beliefs and the dynamic processes engaged in by children, creating meaning within a common cultural framework. This theory maintains that child development is a social process that is impacted by adult intervention and cultural tools (Podolskiy, 2012). In the case of this article, cultural tools are represented in the learning materials and the selected picture book. The application of cultural-historical theory has already been applied in Meng et al.’s (2022) research which argues that a culturally appropriate play plan in China could children’s regulation of fear. This article also applies cultural-historical theory to create a culturally appropriate play plan for anger recognition and management.

Serving as auxiliary theories or supporting models, Piaget’s (1983) constructivist theory which posits that children construct knowledge through interaction with their surroundings is useful when using play. Vygotsky’s (1987) sociocultural theory expands on this by highlighting the role of social interactions, introducing the concept of the Zone of Proximal Development (ZPD), where children learn more effectively with the guidance of a more knowledgeable other. Taking Piaget (1983) and Vygotsky (1987) together, play becomes a vehicle for individual learning and social interaction. This environment, however, must be considered in terms of its placement in the global context. For instance, an ECE setting in China differs from a setting in the US, such as in China’s more centralised, government-controlled structure, as well as societal differences that impact the level of investment for both countries (Hernandez, 2024).

**Figure 1:**
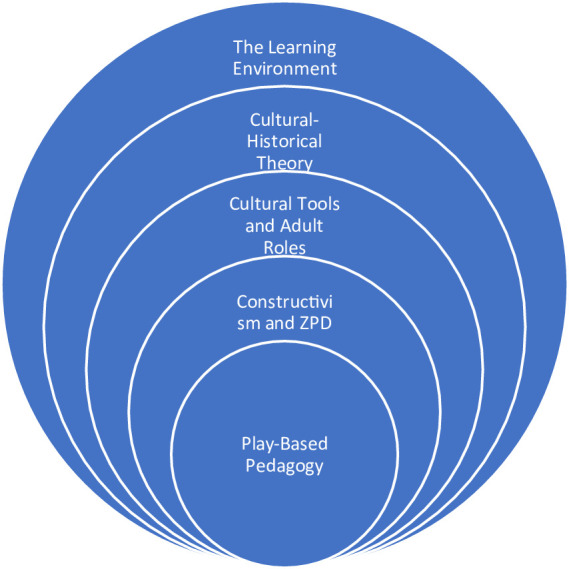
Theoretical framework used to create the weekly plan.

Play was selected as the pedagogical approach because it can support children with their emotional development, especially that related to anger through experiential learning (Vlaicu, 2014; Hart and Booth, 2017). Likewise, interactive play can support children’s ability to recognise other people’s views (Futterer et al., 2022). However, this study could not implement play in its full Western form since a direct transfer of pedagogy into the Chinese context is not appropriate. As noted by Tan and Chua (2015, p. 686), “There exist fundamental cultural differences between Western and Chinese perspectives on the nature and transmission of knowledge that make education policy transfer in China challenging.” As such, this study placed play between the Western views of Piaget (1983) and Vygotsky (1987) and the Confucian view of education which maintains a teacher-centred approach (Leung, 1998). Figure 1 illustrates the article’s theoretical framework used to create the weekly plan.

This study sought to explore whether play can be used to teach curriculum knowledge alongside an understanding of anger in Chinese kindergartens. Since anger can hinder empathy (Eisenberg et al., 1992), it is a key emotion to explore in ECE. It places play between the Western model and the Confucian context of teacher-led learning and embeds the MoE’s (2012) five areas of learning: Language, Social, Cognitive, Arts, and Health. These five areas were viewed in terms of their role in supporting children’s emotional development; in other words, does the teaching of knowledge in the traditional Chinese ECE classroom naturally support children’s emotional development if taught through the medium of play? For example, the MoE (2012) states that language lessons require teachers to convey emotions through “expressions, actions, and an expressive voice.” By linking knowledge with cultural-historical theory and anger as a key emotion felt by children, a framework that aligns with the government’s educational goals and children’s needs while also overcoming barriers faced by teachers could be created. To measure the impact of this framework, the study addresses the following research questions:

1. To what extent can a knowledge-based curriculum underpinned by cultural-historical theory be taught through play-based pedagogy to support children’s understanding of anger?
2. What are the barriers to measuring children’s emotional development (knowledge and understanding of anger) through play in China’s kindergartens?
3. When teaching cultural and historical knowledge through play, how are children impacted in terms of their performance in language, social, cognitive, arts, and health?

To answer the questions, I attended a kindergarten in Northeast China to work with four classes of children aged between 5 and 6 years old. In total, 40 children and four teachers took part. I worked with teachers to create a week-long play-based pedagogy plan that encompassed the curriculum requirements, teacher-led play activities and games, elements of mutually directed play, and four short opportunities for free play. Due to time limitations and an outbreak of seasonal flu in the research setting, resulting in absent children, a one-week intervention was selected as the most suitable for the kindergarten setting. While it would have been useful to create a longer plan to ensure a more longitudinal intervention, it was important to ensure that the research context agreed with the length of time for this intervention, especially during the seasonal flu outbreak. Thus, while this research does not include a baseline assessment or a longitudinal research methodology, its value in assessing the potential impact of a newly created play-based pedagogy for a Confucian context, measured by an oral assessment and teachers’ interview responses, form a basis on which to conduct future studies and further explore the research context.

The plan was designed based on teachers’ perspectives and understanding of play, the provincial textbook “Jilin,” and maintained the kindergarten’s use of discrete timetabled lessons. This was necessary because the use of play needed to be contextually appropriate for the setting (Tan and Chua, 2015). Following the implementation, the teachers conducted an oral assessment with children to measure their emotional skills linked to the feeling of anger. This data was then used to compare the outcomes of the four classes that took part, exploring outcomes linked to each of the five learning areas. From this, I could discover the barriers to supporting children’s emotional development, providing a foundation for future research.

## Methods

### Sampling and participants

Non-probability sampling was used to recruit one kindergarten a large urban kindergarten in Northeast China. This means that this article’s findings are not intended to be generalisable, but rather, a specific sample was targeted to gather information about a specific educational context in China (Cohen et al., 2017). The kindergarten is typical of many in the region, following national curriculum guidelines (MoE, 2012), but struggling to balance the government’s mandate for play-based pedagogy with academic pressures from parents and the local educational system. Data was collected from a total of 40 5-to-6-year-old children from the same kindergarten (Kindergarten 1). In terms of sex/gender, 23 child participants were boys, and 17 participants were girls. The small sample size was due to a seasonal flu outbreak, thus reducing the four classes’ number of students significantly. Therefore, 40 children took part in the study out of the 160 in the entire cohort. Regarding the children who were present, none were excluded from the weekly plan due to any absence during the week; however, only the children who were physically present for the entire week were included in the final sample. Further information about the child participants is presented in Table 1.

**Table 1:** Sample characteristics of the child participants.

Class	Total participants (*n*)	Girls (*n*)	Boys (*n*)
Class 1	10	4	6
Class 2	6	2	4
Class 3	12	8	4
Class 4	12	3	9

In terms of the teacher participants, four teachers from the kindergarten took part. Table 2 provides demographic information for the teacher participants.

**Table 2:** Sample characteristics of the teacher participants.

Teacher ID	Gender	Age	Position	Educational Background	Years in ECE
T1	F	28	Major teacher	Junior college degree	6
T2	F	28	Major teacher	Junior college degree	6
T3	F	31	Major teacher	Junior college degree	12
T4	F	30	Major teacher	Bachelor’s degree	7

### Research positionality

As Bukamal (2022) explains that positionality represents the researcher’s identity, impacting their view of the social world in interpretivist research. My role in this research was primarily as a researcher who collaborated with teachers to create the weekly plan. I supported teachers with my theoretical knowledge, such as how to plan for different play types, alongside my knowledge of policy requirements. Furthermore, as a previous headteacher, I was able to support teachers with curriculum planning while using my own practical experience of working with children in Chinese kindergartens to ensure that the plan was both founded on theory and successfully practically implemented.

### Research design

#### Epistemological stance and methods of data collection

The research represents an exploratory qualitative intervention study which combines a quantitative oral assessment with in-depth qualitative semi-structured interviews (presented in Table 3). Although a single quantitative method has been used, the emphasis in this research rests on teachers’ views following the creation and implementation of a novel, culturally appropriate play-based plan for anger recognition and management. Hence, this study is interpretivist, meaning that individuals’ interpretations and understandings are varied and subjective, whereby people gain meaning from others’ actions (Blumer, 1992). This data was acquired through the use of semi-structured interviews, which were conducted at the end of the study to capture teacher’s perceptions of play-based pedagogy and its impact on children’s knowledge and understanding of anger. The interviews focused on the teachers’ perceptions, and they were asked to evaluate the children’s engagement, any observable changes in their emotional understanding, and the challenges they faced in facilitating the activities. Semi-structured interviews were commonly used in qualitative research, enabling the preparation of questions in advance, thereby aligning with the study’s aims, while also allowing the space for openness and changes (Brinkmann and Kvale, 2018). The combination of semi-structured interviews with teachers with oral assessments also ensured data triangulation, meaning that multiple methods of data collection and analysis were used to strengthen reliability and internal validity (Merriam, 1998).

**Table 3:** Sample interview questions.

Question by theme	Semi-structured interview questions (as a guide)
Play status	Do kindergartens promote play-based pedagogy, and if so, how? What do you think ‘play’ means to children? How would you define play? Do you need to balance play-based pedagogy with traditional academic subjects? If so, how do you do it? What challenges do you foresee in implementing a play-based curriculum? (e.g., policies, cultural norms, parental expectations)
Provision of Play	Do the children in your class use play in their daily activities? If so, when and where do they play? What is your role during play? How do you think play-based pedagogy changes the role of the teacher compared to formal classroom settings? How long does each play session last on average? How often are the play sessions?
Types of Play	Do you use play to teach any of the subjects? If yes, what types of play? (art/music/PE/language/social/free activity) What other methods have you used to teach your previous classes? (Do you use a teacher-centred approach? Do you work in small groups?)
Play Content	How effectively can play help you achieve your teaching goals? How do you view the balance between play and academic learning in the curriculum? What do you teach during the play activities? (Knowledge or skills?)
Teachers’ Perceptions	How would you define successful play-based pedagogy? What aspects of play-based pedagogy do you think are most effective in Chinese kindergarten settings? Would you like to implement different types of play? If so, what type would you like to try? What is your understanding of that type of play, and how do you think we could implement it? What role should teachers have in each type of play? Why?
Children’s Empathy/Anger	What is your understanding of empathy and anger? Can you give me an example of a time when you saw a child demonstrate good empathy skills and or/angry behaviours? Approximately how many children in your class do you think have age-related-expected empathy skills?

The interviews were recorded and transcribed, and the data were analysed using thematic analysis to identify common patterns and themes in the teachers’ responses (Braun and Clarke, 2006). This meant that I followed Braun and Clarke’s (2006) six stages of thematic analysis: becoming familiar with the data; generating initial codes; searching for themes; reviewing themes; defining themes, and finally, writing up. When reviewing the themes, it was important to ensure that the theme fully represented each included code and that the themes made sense without substantial overlap (Maguire and Delahunt, 2017). Furthermore, any further information shared with me by the teachers, such as any written notes on the oral assessment papers, was then used to further explore these thematic findings. From this, the perceived impact of the weekly plan on children’s anger recognition and management could be attained, representing the starting point for future interventions. To exemplify the thematic analysis of teacher interviews, Table 4 presents the coding process which ultimately led to the creation of the main themes. Through researcher collaboration in the data analysis processes, the interrater reliability ensured that the qualitative data were interpreted to produce consistent results (Armstrong et al., 1997).

**Table 4:** Thematic analysis codes and themes.

Codes	Theme
Learning outcomes Children’s demonstration of empathy Teachers’ views/reflections	Positive Perspectives
An activity interpreted as ‘play’	Misunderstandings
Teacher intervention Impact of teacher intervention	Teacher Influence
Verbal expression Behavioural/action expression	Children’s Expressions of Anger
Progress toward measurable outcomes	Performance

#### Creating the weekly plan

**Figure 2:**
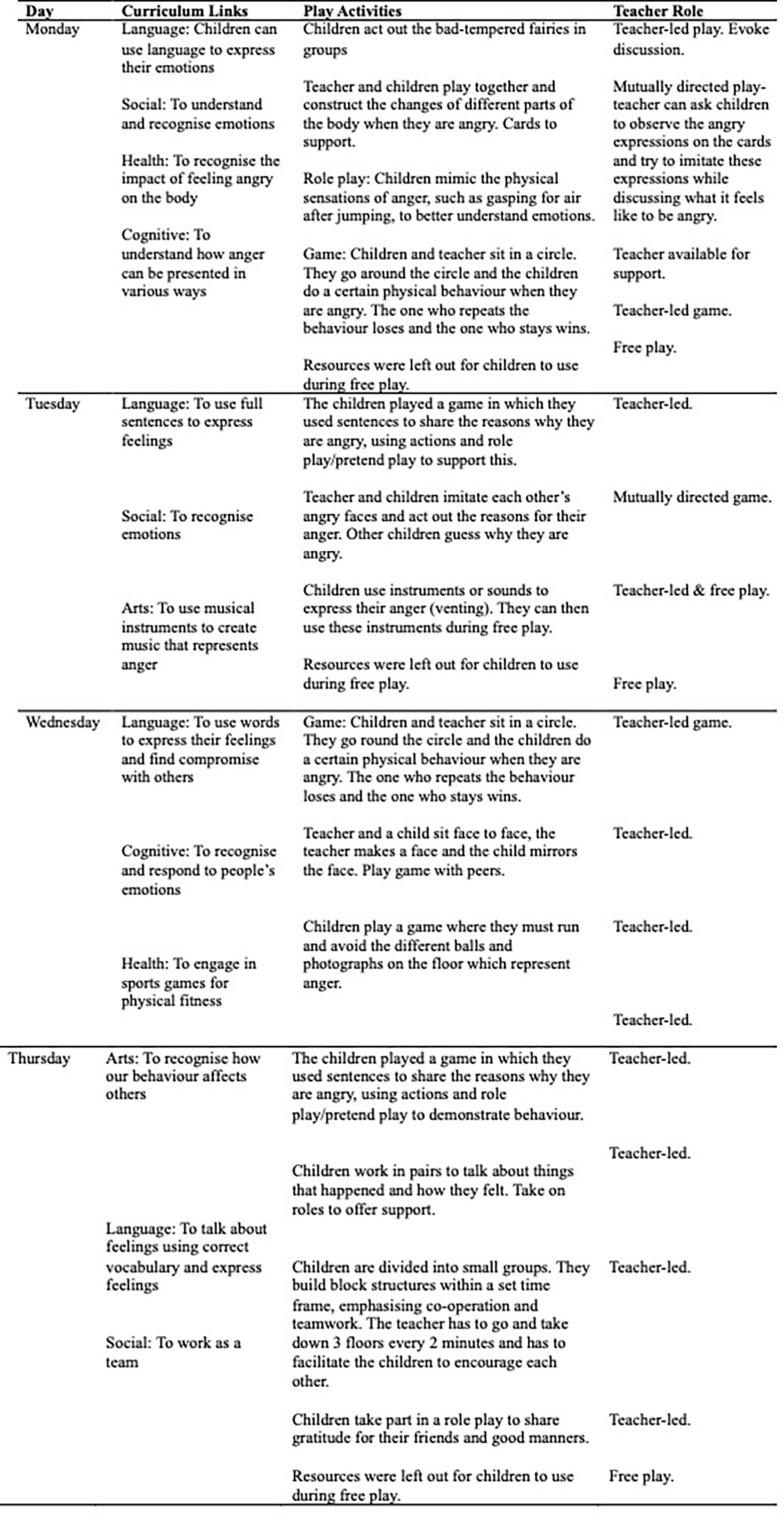
A weekly plan to support children’s knowledge and understanding of anger.

This study used a qualitative approach to explore the integration of play-based pedagogy within a Chinese kindergarten’s curriculum. It was designed to assess the effectiveness of play in fostering children’s knowledge and understanding of anger as an emotion, following the use of a week-long play-based pedagogy plan (see Figure 2).

The weekly plan was underpinned by the picture book *The Bad-Tempered Goblin* (*坏脾气小精灵, 快走开!*) by Cheng (2016). This picture book was selected due to its inclusion of anger and since it was created by child psychologists from the American Psychological Association. The activities encompass a range of play opportunities which were linked to curriculum knowledge, culture and history, and were created in collaboration with the teachers, who shared their ideas and knowledge as part of the process. The cultural-history element of the curriculum and activities included that within collectivism, all children joined in group play activities; meanwhile, they could recognise the group’s response to anger rather than solely their own.

The week began with a reading of the selected picture book, and then, all of the play activities were based on the book. As Strouse et al. (2018) explain, picture books are particularly useful when teaching children about the transfer of knowledge to real-world contexts. Therefore, the play tasks based on this picture book were linked to children’s literacy learning and explored anger in a child-centred way. Niland (2023) also advocates for the use of picture books to inspire play and children’s imagination, while Xia (2011) explains that they can support children’s development and cognition. Hence, the activities included storybook-based emotion recognition tasks, group-based cooperative reflection, and using language to define and describe anger. These activities were embedded into the kindergarten’s regular curriculum, aligning with the existing knowledge-based structure while introducing elements of play that encouraged the exploration of anger. For each of the five areas of learning, the oral assessment questions to measure children’s emotional development were based on the government-prescribed curriculum outcomes. The teachers carried out the oral assessment following my guidance and explanation, and this took place on the Friday, following the activities which were planned for Monday to Thursday of the same week. Hence, only the short-term impact of the intervention can be measured in this research, serving as the starting point for a longer-term intervention which specifically seeks to measure the long-term impacts of such a play-based pedagogy plan.

The activities were designed according to Vygotsky et al.’s (1997) cultural-historical theory, emphasising the role of social interaction. They were structured around the five key areas prescribed by the MoE’s (2012) “Guidelines for the Development of Children Aged 3–6″, which are language, social, cognitive, artistic, and health development. Activities included a role-play where children could express anger and explore strategies for calming down, both individually and as a group. Another activity included playing with musical instruments to express what they perceived to be feelings of anger, and at other times, children could play with all of the week’s resources during free play. The play sessions were designed to last between 25 to 30 min and were embedded within the standard daily curriculum to avoid disrupting the overall learning objectives.

It was important to incorporate different play types during the week to give both teachers and children the opportunity to explore play-based pedagogy. This also allowed for a balance between teacher instruction and authority from Confucianism, mutually directed play to reflect the need for collaboration, as well as free play, a play type which is valued to a lesser extent than guided play (Lin et al., 2021).

#### Cultural adaption of an Oral assessment tool

**Figure 3:**
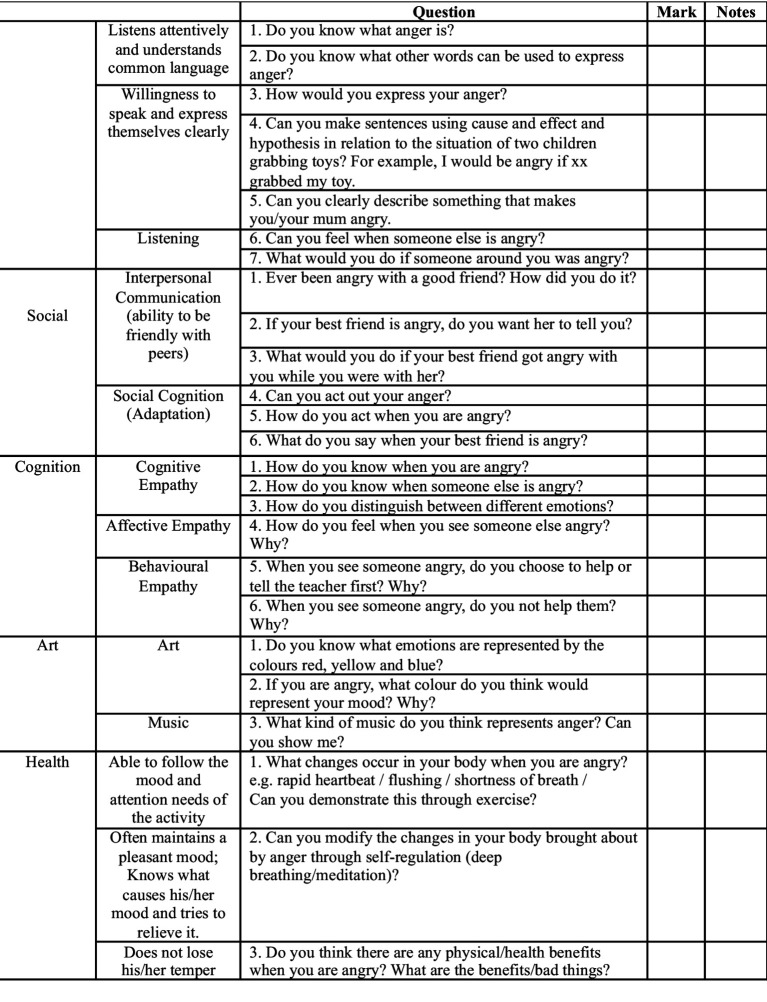
Anger emotion oral assessment.

To complement the qualitative study, an oral assessment was used with children on the final day of the intervention (Figure 3). During the assessment, the children were asked to identify facial expressions and describe scenarios that might evoke feelings of anger. Children were also asked to articulate how they would respond to specific situations. Although not a mixed-methods study, the use of the oral assessment was useful when analysing the qualitative data derived from teachers’ verbal responses, providing a more holistic overview of the research context. Hence, the emphasis of this research remains on subjective understandings and beliefs, rather than taking a positivist epistemological approach (Matthews and Ross, 2010). This was useful in terms of triangulation, whereby the oral assessment data could be used in comparison with teachers’ statements, offering a more rounded view of children’s anger recognition while ensuring research quality (Matthews and Ross, 2010).

To create the oral assessment, the MoE’s (2012) “Guidelines for the Development of Children Aged 3–6” document was used to source the requirements for children’s emotional understanding based on the five domains: language, social, cognitive, artistic, and health development. Aspects of the EmQue-CA as developed by Overgaauw et al. (2017) were also used, most notably in the ‘Cognition’ part of Figure 3, noting that this is a valid scale which can be used to measure children’s affective, cognitive, and behavioural empathy. This is also present in Rieffe et al.’s (2010) EmQue, which divides empathy into these three types. The questions presented in Figure 3 are written in a similar format to Overgaauw et al.’s (2017, p. 6) EmQue-CA, which includes the questions, “When a friend is upset, I am upset too” and “If a friend is sad, I like to comfort him.” Although Overgaauw et al.’s (2017) questions focus on sadness, the format of questioning was applied to Figure 3’s questions about anger, using child-friendly language and including the emotive word ‘anger’. Based on the children’s responses, teachers were asked to award a score from 0 to 4 per question, depending on how closely the answer aligned with expectations. More specifically, 0 would indicate that the child cannot meet the assessment item; 1 would equate to a slight understanding; 2 a moderate understanding; 3 a good understanding, though some gaps may remain; and 4, a good, full understanding. To ensure consistency, I discussed the scoring criteria with the teachers before administration, including examples of responses. The total scores were later analysed descriptively, as outlined in the following section.

An oral assessment was chosen to align with China’s exam-oriented educational system, ensuring that the assessment was culturally appropriate and familiar to the participants. For instance, since 2021, children in China’s primary schools have been orally assessed in their first 2 years (MoE, 2021). Moreover, from age five, the typically developing child in terms of speech and language can use short and simple sentences and define words, meaning that this is a suitable form of assessment (Foster-Cohen, 1999). Teachers were also involved in the assessment creation, using their practical experience to refine the questions and ensure cultural appropriateness.

As discussed earlier in this article, classroom management and assessment are key concerns in China’s ECE settings. This includes large class sizes and limitations in teachers’ confidence and abilities to assess through observation (Zhu, 2007; Wu et al., 2012; Shu, 2022). Moreover, the MoE (2022) does not provide assessment tools to measure children’s social and emotional learning as it prioritises academic assessment (Yu, 2013). Therefore, this oral assessment tool which observes one child at a time does not take too long to administer (this took on average around 15 min per child), merges a familiar assessment technique, suited to the Confucian learning context, with an assessment created to directly measure children’s knowledge and understanding of anger. This gave teachers the opportunity to evaluate the socio-emotional benefits of play, as well as its use in teaching cultural and academic knowledge, thereby aiding their professional development.

#### Data analysis

Descriptive statistics were used to analyse the oral assessment data, meaning that rather than using more inferential statistical models, this research could use the numerical data to complement the teachers’ qualitative interviews. This decision was based on methodological and epistemological considerations. First, the sample size (*n* = 40) is relatively small and does not include random grouping. Thus, the statistical power required for reliable inferences (e.g., ANOVA or t-tests) is insufficient, potentially leading to unreliable results, and observed between-group differences may stem from class composition rather than intervention effects. Second, the oral language assessment is embedded within a larger, culturally contextualised intervention programme and conducted by classroom teachers. This format prioritises context sensitivity and children’s familiarity over psychometric standardisation. Third, the overall objectives of this study are exploratory and interpretative. Descriptive statistics were used to support and contextualise the themes identified in the teacher interviews, thereby providing a more comprehensive understanding of how children’s understanding of anger manifests across different classes and developmental domains. As previously noted, this research is qualitative in its epistemological approach, and thus, the emphasis is not on positivist or statistical perspectives (Matthews and Ross, 2010). Therefore, an inferential analysis of the oral assessment data would not have been appropriate. Future research could conduct inferential statistical analyses by expanding the sample size, setting up a control group, or adopting a pre-post design to further validate the trends identified in this exploratory phase.

#### Gender comparisons

Glasser and Smith (2016) explain that it is necessary to define the meaning of ‘gender’ in a piece of research; hence, the term ‘gender’ in this article refers to children’s sex as shared by their class teacher. I decided to distinguish between scores by boys and girls because, during the data analysis, I saw that there were differences between groups. This provides a foundation for future research which could explore whether gender stereotypes or roles come into play in terms of play opportunities and resource selection.

### Ethics statement

The study adhered to the ethical guidelines for research, ensuring that all participants and their guardians provided signed informed consent. Parents were informed of the study’s intentions and methods, and throughout the research, I held the view that research with children is essential to improving their own lives and those around them (Fraser et al., 2004). The children’s well-being was prioritised throughout the study, and I ensured that all activities were age-appropriate, safe, and enjoyable. As Farrell (2005) explains, research with children must be done in a safe space where children are respected. The teachers were also briefed about the ethical considerations of the study, including the importance of fostering a supportive and inclusive environment. The institution overseeing this study approved the research before it began. I also followed BERA’s (2024) *Ethical Guidelines for Educational Research*. Furthermore, the teachers and kindergarten administrators signed consent forms to take part. All participation from children and adults was voluntary; they were informed that they could withdraw at any point without consequence.

## Results

The results from the oral assessments with children and teacher interviews provide comprehensive insights into how play-based pedagogy underpinned by cultural-historical theory could be implemented into a knowledge-based curriculum. The data from the oral assessments and semi-structured interviews are presented in this section. For each of the participants, pseudonyms have been used. For example, T1 refers to Teacher 1, T2 for Teacher 2, T3 for Teacher 3 and T4 for Teacher 4. Structurally, the results are presented in table format, linking the interviewees’ responses to the thematic analysis, before being discussed in the following section.

From the thematic analysis, the interview data were analysed to create the themes ‘Positive Perspectives’, ‘Misunderstandings’, ‘Teacher Influence’ and ‘Children’s Expressions of Anger’ (see Table 5). A further theme ‘Performance’ also arose from the interview data; however, this is shared after Table 5 since it is used to complement the quantitative data from the oral assessments.

**Table 5:** Qualitative findings from the interviews.

Theme	Code	Interviewee response
Positive Perspectives	Learning outcomes	“Now the children can proactively speak. Based on their drawings, they can explain what they have created, which exercises their language abilities. They can also talk to their peers about their work, improving interpersonal communication skills. This storytelling activity has helped enhance many abilities.” (T1). “Children have made noticeable progress, particularly in the language domain. For instance, when we interviewed them, they were able to recount stories about their mothers or personal experiences in detail.” (T1). They [the children] can now retell a complete story, explaining why they were upset, and accurately describe their feelings using appropriate words. In the social domain, they are better able to experience and understand different emotions and the effects those emotions have. (T2).
	Children’s demonstration of empathy	When one child knocked over another’s cups during play, another child observed and explained, ‘He’s upset because you knocked over his cups.’ (T3).
	Teachers’ views/reflections	T4 shared their view that play-based pedagogy largely met their expectations, stating “This approach met about 80% of my expectations for play-based pedagogy. The remaining gaps are due to our own lack of experience.” I feel much better because with [the researcher’s] guidance, we have discussed and collected information together. At least most of the children can experience various emotions through play and learn how to release them. I think it’s good to know what to do when children throw tantrums. (T3).
Misunderstandings	An activity interpreted as ‘play’	One teacher believed that reading a story is a play activity. When tasked with designing play activities aimed at fostering empathy or feelings of anger, many teachers lacked originality and resorted to using fragmented game ideas sourced from social media.
Teacher Influence	Teacher intervention	T4 shared a time when they worked with two children who were stomping their feet onto each other’s feet in the classroom. The children were presenting this behaviour when the other members of the class were listening to the teacher’s input. To deal with this, T4 asked: “What are you doing?,” then shared with me “Maybe my tone of voice changed suddenly, and the two children did not say a word, did not respond in any way, did not answer my question, and did not say why they were stepping on each other’s feet. But after that, I asked them. After that, the children went to a different time slot, and it might have been a long time. I asked again, saying, ‘Why were you stomping your feet during class just now?’” (T4). “If we do not guide the children, they often do not know how to start the play activities on their own.” (T1) T2 shared that when children were angry, they would “appropriately assist them [the children].”
	Impact of teacher intervention	T4 explained that they believed the way that they dealt with children’s actions may have led to them feeling criticized, and therefore, unwilling to share their feelings.
Children’s Expressions of Anger	Expressing Anger	“Many children understand what it means to be angry, but they do not know how to express it in words.” (T2)

Overall, teachers noted improvements in children’s empathy following the short intervention, suggesting that children had gained the necessary vocabulary and emotional skills related to anger, thereby potentially reducing instances of angry outbursts (Strayer and Roberts, 2004). However, the misunderstandings clearly suggest that further interventions or professional development in the research setting is needed to fully support teachers with their understanding of play-based pedagogy.

Turning to the quantitative data and qualitative theme ‘Performance’, Table 6 presents the results of the oral assessment.

**Table 6:** Results from the oral assessments (quantitative data).

Class	Domain	Average score (Boys)	Average score (Girls)	Difference in scores	
				Boys	Girls
Class 1	Overall	56.5	70.5	-14	+14
	Language	17.16	17.25	−0.09	+0.09
	Social	15.16	19.25	−4.09	+4.09
	Arts	6.5	8.5	−2	+2
	Cognitive	14.5	15	−0.5	+0.5
	Health	4.5	7.25	−2.75	+2.75
Class 2	Overall	75.75	78	−2.25	+2.25
	Language	21.75	23	−1.25	+1.25
	Social	20.75	23	−1.25	+2.25
	Arts	8.5	7	+1.5	−1.5
	Cognitive	17.5	17	+0.5	−0.5
	Health	6.75	8	−1.25	+1.25
Class 3	Overall	77	79.37	−2.37	+2.37
	Language	21.25	20.75	+0.5	−0.5
	Social	21.75	21.5	+0.25	−0.25
	Arts	10	10.5	−0.5	+0.5
	Cognitive	16.5	16.25	+0.25	−0.25
	Health	9.5	9.75	−0.25	+0.25
Class 4	Overall	57.22	58.33	−1.11	+1.11
	Language	17.11	18.66	−1.55	+1.55
	Social	12.66	11	+1.66	−1.66
	Arts	6.88	8	−1.12	+1.12
	Cognitive	13.11	12	+1.11	−1.11
	Health	5.44	6	−0.56	+0.56

See Table 1 for *n* by class/sex.

All four classes had reasonable scores in the language domain. For instance, Class 3 had the highest language score at 20.5 out of a possible 28, while on the contrary, Class 1 scored 16.6. Thus, discrepancies in scores arose between classes. However, teachers commented on overall improvements due to the implementation, including T1, who mentioned that “The children have improved compared to before. For example, in the language area, the children have reached a level where they can communicate,” and T2 explained that:

They can retell a story in its entirety, that is, tell a story about themselves, and then be able to tell why they are not happy, what is not happy, and be able to describe it in words accurately. Then in the social domain, they can experience different emotions and the different feelings they bring. (T2).

Also, T4 mentioned that the children had improved due to the implementation of play-based pedagogy, explaining that children’s listening had improved, and their lack of complaints to the teacher had reduced. Instead, children appeared to be dealing with their own problems in the classroom.

Social scores were relatively high across the sample. Class 3 performed the best in this domain, with an average score of 21.58 out of 24, while Class 4 lagged behind at 13. This discrepancy in scores suggests that while most children benefitted from play-based social interactions, certain classrooms, such as Class 4, may require more targeted interventions to achieve similar results.

Class 3 excelled in the arts, scoring an average of 10.25 out of 12, while Class 1 scored 7.2. The strong performance in this domain reflects the effectiveness of using arts-and-play-based activities to develop emotional awareness and expression in children.

The cognitive scores were highest in Class 2 and Class 3, with averages of 17.33 and 16.33 out of 24, respectively. Class 4 again showed a weaker performance in this area, scoring 13.08. These results further suggest that the play-based pedagogy had a positive impact on children’s cognitive abilities, though variability between classrooms points to the need for more consistent implementation across teachers.

Class 3 outperformed other classes in health, with an average score of 9.75 out of 12, while Class 4 scored 5.83. These results indicate that physical activities, when linked with emotional education, can effectively contribute to children’s overall health development.

From the interviews, the theme ‘Performance’ arose. To begin, language development was frequently highlighted as one of the most noticeable areas of improvement. T1 specifically mentioned how children had shown progress in articulating their thoughts: “Children have made significant progress in the language domain. For instance, when we interviewed them, they were able to recount stories about their mothers or their own experiences in great detail.” Similarly, T2 noted that children could “retell a complete story, explaining why they were upset, and accurately describe their feelings using appropriate words.” These comments indicate that the play-based activities, which involved role-playing and storytelling, were highly effective in enhancing the children’s language skills, including using them to describe their own emotions in a structured, supportive environment.

For social development, teachers frequently commented on children’s abilities to engage in meaningful social interactions. T1 observed, “Children are now able to talk to each other more, share their work, and ask questions about their peers’ activities. This has improved their interpersonal communication skills.” Additionally, T2 remarked on the children’s growing ability to navigate emotional situations with their peers: “In the social domain, they are better able to experience and understand different emotions and the effects those emotions have.”

Arts development and cognitive skills were highlighted by T4, who discussed how art activities, such as drawing and role-playing, allowed children to externalise their understanding of emotions. She noted:

Through role-playing play, children’s drawing skills developed significantly. When they draw, they think about what being angry looks like, and they can represent this emotion through their artwork. It’s impressive because when they accurately record the emotion, they can truly understand it. (T4).

This demonstrates how arts, integrated with emotional learning, can enhance both creative and cognitive skills in young children.

Health development was measured through activities that focused on physical engagement and emotional understanding. According to T4, the physical aspects of the play helped children to “understand what it feels like to be angry, both mentally and physically. They moved their bodies and felt what it’s like to express emotions through action.” This integration of physical and emotional learning fostered a holistic approach to health education.

## Discussion

### To what extent can a knowledge-based curriculum underpinned by cultural-historical theory be taught through play-based pedagogy to support children’s understanding of anger?

The data from the four classes’ oral assessments (Table 6) shows that Class 4’s average scores were lower than the other classes, while Classes 2 and 3 have the highest scores. More specifically, three out of the four classes (Classes 1, 2 and 3) scored above 60 in the oral assessment, indicating potential success of the play activities for the majority of children. Class 2, for instance, had an average score of 76.5, and Class 3 scored 78.5. These scores exceeded teachers’ initial expectations, validating the approach’s efficacy in enhancing learning in various developmental domains, particularly language and social skills. On the contrary, Class 4, had an average score of 56.83, lagging behind the other classes. This shows that there are discrepancies in children’s outcomes at the end of the intervention, bringing into question whether the approach is suitable for all children, or whether some teachers require more support than others in implementing a play-based approach. The latter is a high possibility, especially when considering that there are inconsistent approaches to using play in China’s ECE settings (Zhu and Zhang, 2008). Such an inconsistency is exemplified in this data, and was commented on by one of the teachers, who shared that, “Our lack of experience is one of the reasons behind the uneven scores across classes.” (T4).

This sentiment was shared by several teachers, who expressed a need for further guidance and training to fully integrate play-based learning with traditional academic objectives. Interestingly, the T4’s use of the word ‘our’ in this case demonstrates the ingrained view of collectivism in the Chinese education system (Wang, 2006), even in the cases when the ‘lowest-performing’ class teacher was discussing differences in the data. Moreover, the teachers all shared their views that the children had improved in terms of their knowledge and understanding of anger as an emotion. Hence, according to the teacher, Class 4 will have had particularly low levels of empathy and emotional awareness at the start of the study, meaning that they were at risk of higher levels of anger or aggressiveness (Strayer and Roberts, 2004).

Discrepancies in attainment could also be explained by a range of factors, such as children’s overall attainment (Eisenberg et al., 2013; Oberle et al., 2014), teachers’ involvement and level of engagement in the play activities (Fleer, 2018), the teaching content (Hedges and Cooper, 2016; Edwards, 2017) and the age of children (Lewis and Granic, 2010; Uzefovsky and Knafo-Noam, 2016). Moreover, taking the findings from 5.3, teachers’ understanding of child development and children’s ability to use words to express their feelings could impact children’s assessment results on the oral test, especially if they are not yet at the age-related expectation for five-year-olds, which includes using short and simple sentences and defining words (Foster-Cohen, 1999).

The quantitative data show that the oral assessment scores differed between genders. Studies have already demonstrated that boys show more aggression and anger than girls and have lower levels of empathy (Strayer and Roberts, 1997; Coie and Dodge, 1998). However, the types of empathy that boys have lower levels of appear to be overlooked to an extent, despite reiterating the link between empathy and anger in the early years. In this study, it was found that there were clearly gendered affordances of play, with Class 1’s girls having an average score of 70.5 compared to boys’ 56.5; and in Class 4, girls’ language score was 18.66 while it was 17.11 for the boys. Moreover, in Class 4, boys’ cognitive score was 13.11 while it was 12 for the girls. This correlates with previous research findings which note that girls tend to outperform boys in early language development (Tse et al., 2002; Eriksson et al., 2012; Lange et al., 2016), while boys outperform girls in cognitive paper folding tasks (Huang, 1993). These outcomes suggest that boys and girls may engage and benefit from play in different ways, pointing to the need for differentiated instructional strategies. As a result, it is essential to consider how to include all genders in terms of resource selection and play opportunities in the Chinese context.

In summary, play can be used to support a knowledge-based curriculum, and through culturally appropriate play activities, cultural-historical theory aligns with the current Chinese kindergarten curriculum. Nonetheless, some challenges to implementation are apparent, especially when comparing data from each of the four classes, which highlights potentially inconsistent approaches in the classrooms (Zhu and Zhang, 2008).

### What are the barriers to measuring children’s emotional development (knowledge and understanding of anger) through play in China’s kindergartens?

It can be summarised that the use of play activities in the weekly plan is best viewed as a transitional or temporary solution. Although it has been found that play-based pedagogy is effective in fostering children’s language, social, and emotional development with regard to anger, as highlighted by teachers in their reflections and in the quantitative data, the extent to which play can improve children’s knowledge and understanding of anger is dependent on teachers’ understanding of play. Hence, this approach may only serve as a temporary or stop-gap measure until teachers are trained to create broader play opportunities which are more child-led, such as free play.

Despite that the weekly plan (Figure 2) temporarily satisfies policy demands for play-based learning and parental expectations for academic outcomes, it does so by designing the curriculum as a series of isolated, independent activities rather than as part of a cohesive and integrated framework. This does, however, reflect China’s curriculum plans that align with structured, teacher-led activities (Leung, 1998). The lack of cohesion and consistency in curriculum design could lead to diminished outcomes, as children may struggle to transfer their knowledge and skills about anger acquired in one play-based activity to other areas of learning.

Finally, the integration of Western ideas of play into a Confucianist society which upholds collectivism may actually challenge the notion of the importance of individual teachers reflecting on their own practice. In the case of T4, who referred to “Our lack of experience” rather than ‘my lack of experience’ shows a collaborative and collective approach to teaching (Huang, 2006), even in the case of T4’s class performing at a lower level than the other classes. Therefore, to ensure that play-based pedagogy is used effectively, collectivism can be maintained to ensure consistency, yet individual professional development targets are vital (Darling-Hammond et al., 2017). Hence, a balance between collectivism and individual reflection needs to be implemented. In practice, this may mean implementing such methods as Professional Learning Communities (PLCs), whereby teachers can collaborate to improve their teaching and students’ learning (Fullan, 2003). Since PLCs are present in China in the form of a top-down institutional requirement for such tasks as lesson planning (Chen, 2006), these can be applied when planning play activities; however, it would be useful to allow teachers to meet on occasions without school leaders, allowing reflection to take place without the authoritarian element. As Zhang and Pang (2016) explain, teachers in China require power and responsibility to conduct their own collective learning activities which can be both spontaneous and informal in their nature (Zhang and Pang, 2016). Thus, reflection and collective collaboration through informal PLCs may support teachers with their implementation of play-based pedagogy.

### When teaching cultural and historical knowledge through play, how are children impacted in terms of their performance in language, social, cognitive, arts, and health?

The use of this weekly plan has demonstrated positive effects on children’s development across several key domains, particularly in language, social, and arts development. Teachers observed notable improvements in these areas, and the data from the oral assessments corroborate these findings. This indicates that the designed play-based pedagogy is effective in supporting the development of key communication and interpersonal skills as the result of exploring anger in this research setting. The arts domain also benefited from this approach, with children showing greater creativity and emotional expression through art-based activities. This concurs with Alavinezhad et al.’s (2014) research in the field of art therapy, whereby art is an appropriate method in which to explore feelings. Hence, this is a key finding when seeking to integrate play-based learning into Chinese kindergartens. Thus, the use of play-based art activities provides space for a teacher-planned activity where children have the freedom to select art materials, play with these materials, and express their emotions creatively. When considering that the results of this research found that girls generally outperformed boys in the language and arts domains, teachers can provide boys in particular with such creative, playful experiences to fill this gap. As previously discussed, boys’ empathy and their empathetic behaviours tend to be lower than girls, something which continues into adulthood (Pifer et al., 1994; Leppänen and Hietanen, 2001; Chapman et al., 2006; Daly and Morton, 2006). Interestingly, it was also discovered that boys showed a slight advantage in cognitive and social development learning areas. This would certainly be an interesting scope for future studies which can discover if this is a common trend, and if so, why boys’ overall empathy levels appear lower than girls.

Finally, the global implications of this research are exemplified by the beneficial use of play and creative activities for supporting children’s knowledge and management of anger. While positioned in a specific Chinese context, children’s feelings of anger can be generalisable to other contexts. For instance, in other areas of Asia where Confucianism drives teacher-led learning, the introduction of some play-based learning for anger management and empathy could be trialled. Likewise, even in the West, countries such as the US can integrate creative, art-and-play-based learning tasks to help develop boys’ empathy and anger management, helping to bridge the gender gap. This is of critical importance given the impact that hegemonic masculinity and bullying can have when students are not equipped with the appropriate skills to manage their anger (Rosen and Nofziger, 2019).

## Conclusion

This research investigated whether a carefully designed weekly plan which uses play-based pedagogy to support anger recognition and management can be effectively used in a knowledge-based curriculum context (China). The findings provide valuable insights into the challenges and potential of using play to support children’s knowledge and understanding of anger, an emotion which can impact their wider emotional, cognitive, and social development. The findings also highlight the limitations of the current implementation in the Chinese context.

To begin, play can be used as part of a knowledge-based curriculum by ensuring cultural-historical theory is applied. In practice, this means creating culturally relevant learning scenarios that teach children the knowledge required to define and recognise anger, as well as the skills to manage their behaviours. However, the weekly plan designed and implemented in this research is only a short-term solution. This is because teachers in Chinese kindergartens may not fully understand how to use play, and inconsistencies in its usage can be present across different classes in the same setting. As such, professional development is required, possibly at the initial teacher training level, in addition to curriculum refinement which encompasses skills-based outcomes. Moreover, in terms of assessment, the oral assessment used in this research was useful in measuring children’s abilities to recognise and discuss anger, yet this also needs further testing and usage to determine whether such an assessment can be generalised to other research contexts. Furthermore, future research should consider using baseline assessments on which to compare assessment scores both before and post intervention, as well as applying a more longitudinal approach to fully analyse the impact of such an intervention. Nonetheless, for this research, the data derived from the oral questionnaire was useful in comparing gender-based outcomes in the five areas of learning, most notably in the importance of giving boys access to creative and art resources to provide a physical space for emotional expression. This is of crucial importance given the impact that anger can have on the behaviours associated with hegemonic masculinities later in adolescence (Rosen and Nofziger, 2019).

It is essential to ensure that any interventions are culturally appropriate to the research context, and therefore, although the weekly plan and oral assessment can be used in other contexts, they must be adapted to specific ECE settings through teacher and leadership collaboration, whereby the sessions and assessment can be further refined. Nonetheless, given that anger continues to be a worldwide issue considered from many research perspectives, as demonstrated in this article’s introduction, the impact that this plan had on the specific research setting offers an optimistic foundation for future studies in other global contexts.

Limitations of this research include the omission of a baseline assessment and randomisation. In future studies, a baseline assessment would be beneficial for data comparison within a longitudinal study, whereby teachers are supported with integrating play-based pedagogy for anger management over, for example, an academic year. Furthermore, this research only reports on descriptive statistics, and thus, future research could strengthen this study by adopting pre-post comparisons with a control and test group design. Finally, the oral assessment requires further refinement and testing to determine its generalisability. Nonetheless, its use in this research was useful in its purpose of collecting data and providing an overview of the current situation regarding empathy and anger management in the research setting.

## Data Availability

The datasets presented in this article are not readily available because the datasets generated and analyzed during the current study are not publicly available due to confidentiality and privacy agreements established with participants and the participating institution, but are available from the corresponding author upon reasonable request. Requests to access the datasets should be directed to Qiming Liu, ql310@cam.ac.uk.
